# Bidirectional field-steering and atomic steering induced by a magnon mode in a qubit-photon system

**DOI:** 10.1038/s41598-023-41907-7

**Published:** 2023-09-11

**Authors:** Ahmed A. Zahia, M. Y. Abd-Rabbou, Ahmed M. Megahed, A.-S. F. Obada

**Affiliations:** 1 Department of Mathematics, Faculty of Science, Benha University, Benha, Egypt; 2 Mathematics Department, Faculty of Science, Al-Azhar University, Nasr City, Cairo, 11884 Egypt

**Keywords:** Quantum information, Qubits, Single photons and quantum effects, Theoretical physics

## Abstract

This paper investigates the cavity–magnon steering and qubit–qubit steering of a hybrid quantum system consisting of a single-mode magnon, a two-qubit state, and a single-mode cavity field in the presence of their damping rates. The temporal wave vector of the system is obtained for the initial maximally entangled two-qubit state and initial vacuum state of the magnon and cavity modes. Additionally, the mathematical inequalities for obtaining the cavity–magnon steering and qubit–qubit steering are introduced. The findings reveal that steering between the magnon and cavity is asymmetric, while steering between the two qubits is symmetric in our system. Increasing the atom–field coupling improves steering from magnon to field, while reducing steering between the two qubits. Moreover, increasing magnon–field coupling enhances and elevates the lower bounds of qubit–qubit steering. Further, adding the damping rates causes deterioration of the cavity–magnon steering and qubit–qubit steering. However, the steering persistence is slightly greater when damping originates from the cavity field rather than the magnon modes based on the coupling parameters.

## Introduction

In the majority of quantum optics tools, the two-level system and the harmonic oscillator are two fundamental models. The Rabi model^1^ and the Jaynes–Cummings (JC) model^2,3^ are two well-studied models that arise from combining these two models into a bipartite system . The Rabi model was initially developed for NMR systems, while the JC model was introduced to describe the interaction between a two-level atom and a quantized electromagnetic (EM) field. The JC Hamiltonian can be derived from the Rabi Hamiltonian by imposing the rotating wave approximation (RWA)^4^. However, in certain circumstances, the JC model is exact, and the RWA terms naturally vanish. Despite its simplicity, the JC model has accurate analytical solutions. Its dynamics have proven to be remarkably intricate and diverse, encompassing a wide range of physical phenomena. These include anti-bunching^5,6^, collapse-revivals^7,8^, squeezing^9,10^, quantum phase transition^11,12^, atom–field entanglement^13^, and non-classicality^14,15^. Furthermore, a variety of other systems, including trapped ions^16^, Cooper-pair boxes^17^, “flux” qubits^18^, and Josephson-junctions^19^, have demonstrated the applicability of the JC model, which was originally conceived as a model for a single atom interacting with a single field mode.

Recently, the generalization of field–atom interaction has presented a new type of hybrid quantum system which exhibits new and interesting properties that have not been presented in either of the constituent quantum systems^20^. One of the most popular hybrid quantum systems is studying the new cavities associated with magnons (including: magnetic dipole^21,22^, magneto-optical^23,24^, and magnetostrictive interactions^25^), micromechanical resonators^26^, and LC resonators^27^. In general, hybrid quantum systems based on magnon provide opportunities to create cutting-edge quantum technologies, such as microwave-to-optical quantum transducers^23^ for processing quantum information and quantum-enhanced detection of magnons for uses like magnon spintronics^28^ and dark matter searches^29^. Considering decay and decoherence in quantum systems is useful for providing a more accurate model of real physical systems, where the quantum systems interact with their surrounding environment^30,31^. Decay and dissipation affect the quantum correlation between components in hybrid quantum systems like cavity/circuit QED, and magnon–cavity systems, and quantum dot system^32–35^.

The concept of quantum steering was first introduced by Schrödinger in the context of an investigation on the Einstein–Podolski–Rosen (EPR) paradox^36,37^. The aforementioned concept pertains to the capacity of an observer to impact the situation of a remote system using local observations. Specifically, if Alice and Bob are in a state of entanglement, Alice can manipulate Bob’s state from a distance by solely conducting measurements on her half of the system. Such an action is unfeasible if the shared state is only classically correlated. This form of quantum correlation is commonly known as EPR steering. Entanglement, the EPR paradox, and Bell’s nonlocality were all thought to need the same resources until Werner^38^ found that not all entangled states violate a Bell inequality. Steerable states can be used in several disciplines of quantum information theory^39^. EPR steering is a powerful tool for determining quantum correlations. It has been used in a variety of practical applications, including: the viability and security of a one-sided device for standard quantum key distribution protocols^40^. Additionally, it has been used for quantum teleportation security and quantum computation^41,42^. Quantum steering has also been studied for some quantum systems, such as the bipartite two-qubit X-state^43^, Heisenberg chain models^44^, and two- or three-level detectors^45^. It is vital to mention how quantum system’s parameters affect the steering process because quantum systems constantly interact according to their parameters.

The primary objective of this paper is to investigate the possibility of steering between two distinct mode fields, namely cavity and magnon, and to determine whether a steered correlation can be generated between them despite their initial separable state and weak interaction. We aim to determine whether the interaction between the field and the magnon is symmetrical or whether the interaction of the field with the atom alters the steering symmetry. To this end, we will introduce a mathematical correlation for field steering between these two different modes using probabilities for joint measurements and a Local Hidden State (LHS) state^46,47^. Furthermore, we intend to examine the impact of the magnon–field interaction on atomic steering. Specifically, we will investigate whether this interaction reduces or increases atomic steering, even though the initial state has a maximum value of steering (i.e., Bell state). Additionally, we will analyze the effect of various system parameters, including detuning and coupling parameters, on both atomic and field steering. So, the paper is organized as follows: in “Structure of the Hamiltonian system”, we described our hybrid physical system and exact solution using time-dependent Schrödinger equation. “Steering inequality” is devoted to demonstrating the steering of the two-mode field and EPR-steering of an atomic system, additionally, we presented some numerical results to discuss steering inequalities. Finally, we summarize our findings.

## Structure of the Hamiltonian system

Let us consider a hybrid Hamiltonian system that consists of two two-level atoms (two-qubit) and a bosonic magnon mode inside a microwave cavity field. We ignore the interaction between qubits and magnon mode, and adding the magnon, cavity and qubit decay to the Hamiltonian model. The non-Hermitian Hamiltonian of this system can be written as $( \hbar =1 )$^48,49^

1 $$\begin{aligned} \hat{H}= & \,\omega _c\hat{c}^\dagger \hat{c}+ \omega _m\hat{m}^\dagger \hat{m}+\sum _{j=1}^{2}\bigg (\frac{\omega _q}{2}\hat{\sigma }^{(j)}_z+\lambda _1\hat{\sigma }^{(j)}_x (\hat{c}+\hat{c}^\dagger )-i \frac{\gamma }{2}\hat{\sigma }_+^{(j)} \hat{\sigma }_-^{(j)}\bigg )\nonumber \\{} & {} +\lambda _2 (\hat{c}+\hat{c}^\dagger )(\hat{m}+\hat{m}^\dagger )- i\frac{\kappa _c}{2} \hat{c}^\dagger \hat{c}- i\frac{\kappa _m}{2}\hat{m}^\dagger \hat{m}, \end{aligned}$$

where $\hat{c}$, $\hat{m}$, $\hat{c}^\dagger$, and $\hat{m}^\dagger$ are the annihilation (creation) operators of microwave cavity mode and magnon mode, respectively, with frequencies $\omega _c,\omega _m$. $\hat{\sigma }^{(j)}_z =|e\rangle _{jj}\langle e|-|g\rangle _{jj}\langle g|$, $\hat{\sigma }^{(j)}_x =|e\rangle _{jj}\langle g|+|g\rangle _{jj}\langle e|$, $\hat{\sigma }^{(j)}_+=|e\rangle _{jj}\langle g|$ and $\hat{\sigma }^{(j)}_-=|g\rangle _{jj}\langle e|$ are the Pauli spin operators of the qubit system and $|e\rangle$ ($|g\rangle$) is the excited (ground) state of the qubit $i=1,2$. $\lambda _{1}$, ($\lambda _{2}$) are the effective coupling strength of microwave cavity–qubit interaction (cavity–magnon interaction). $\gamma$, $\kappa _c$, and $\kappa _m$ are the decay rates of the two-qubit, cavity field, and magnon, respectively.

Applying the RWA in the frame of the interaction picture under the resonance condition $\omega _q=\omega _m$, we get^50^

2 $$\begin{aligned} \hat{H_I}=\lambda _1 \left( e^{i \delta t} \hat{c} \sum _{j=1}^{2} \hat{\sigma }_+^{(j)} +h.c. \right) + \lambda _2 \big (e^{i \delta t}\hat{c}\hat{m}^\dagger + h.c\big )-i \frac{\gamma }{2} \sum _{j=1}^{2} \hat{\sigma }_+^{(j)} \hat{\sigma }_-^{(j)}- i\frac{\kappa _c}{2} \hat{c}^\dagger \hat{c}- i\frac{\kappa _m}{2}\hat{m}^\dagger \hat{m}. \end{aligned}$$

To obtain the temporal state vector under investigation, we begin by determining the initial state vector. Specifically, we assume that the two-qubit system is initially in the Bell state $|\psi _q\rangle = \frac{|eg\rangle +|ge\rangle }{\sqrt{2}}$, while the cavity field and magnon are both initially in vacuum states. Therefore, one can write the initial state vector of our system as

3 $$\begin{aligned} |\psi (0)\rangle =\frac{|eg0_c,0_m\rangle +|ge0_c,0_m\rangle }{\sqrt{2}}. \end{aligned}$$

Under the action of interaction Hamiltonian (2) and initial state (3), the state vector $|\psi (t)\rangle$ reads

4 $$\begin{aligned} |\psi (t)\rangle =c_1(t)|g,g,0_c,1_m\rangle +c_2(t)|g,g,1_c,0_m\rangle +c_3(t)|e,g,0_c,0_m\rangle +c_4(t)|g,e,0_c,0_m\rangle , \end{aligned}$$

where, $\sum _{i=1}^{4}|c_i (t)|^2=1$. Employing the time-dependent Schrödinger equation $i\frac{\partial {|\psi (t)\rangle }}{\partial {t}}=\hat{H_I}|\psi (t)\rangle$ and using Eqs. (2, 3), the exact probability amplitudes $c_i (t)$ can be obtained as

5 $$\begin{aligned} c_1(t)= & {} \sqrt{2} \lambda _1 \lambda _2 \frac{ \eta _1 \left( e^{\eta _2 t}-e^{\eta _3 t}\right) +\eta _2 \left( e^{\eta _3 t}-e^{\eta _1 t}\right) +\eta _3 \left( e^{\eta _1 t} -e^{\eta _2 t}\right) }{\left( \eta _1-\eta _2\right) \left( \eta _1-\eta _3\right) \left( \eta _2-\eta _3\right) },\nonumber \\ c_2(t)= & {} -i \sqrt{2} \lambda _{1} e^{i\delta t}\bigg (\frac{e^{\eta _1 t} \left( \eta _1+\frac{\kappa _m}{2}\right) }{\left( \eta _1-\eta _2\right) \left( \eta _1-\eta _3\right) }+\frac{e^{\eta _2 t} \left( \eta _2+\frac{\kappa _m}{2}\right) }{\left( \eta _2-\eta _1\right) \left( \eta _2-\eta _3\right) }+\frac{e^{\eta _3 t} \left( \eta _3+\frac{\kappa _m}{2}\right) }{\left( \eta _3-\eta _1\right) \left( \eta _3-\eta _2\right) }\bigg ),\nonumber \\ c_3(t)= & {} \frac{e^{\eta _1 t} \left( \lambda _2^2+\left( \eta _1+\frac{\kappa _m}{2}\right) \left( \frac{\kappa _c}{2}-i \delta +\eta _1\right) \right) }{\sqrt{2}\left( \eta _1-\eta _2\right) \left( \eta _1-\eta _3\right) }+\frac{e^{\eta _2 t} \left( \lambda _2^2+\left( \eta _2+\frac{\kappa _m}{2}\right) \left( \frac{\kappa _c}{2}-i \delta +\eta _2\right) \right) }{\sqrt{2}\left( \eta _2-\eta _1\right) \left( \eta _2-\eta _3\right) }\nonumber \\{} & {} +\frac{e^{\eta _3 t} \left( \lambda _2^2+\left( \eta _3+\frac{\kappa _m}{2}\right) \left( \frac{\kappa _c}{2}-i \delta +\eta _3\right) \right) }{\sqrt{2}\left( \eta _3-\eta _1\right) \left( \eta _3-\eta _2\right) },\nonumber \\ c_4(t)= & {} c_3(t), \end{aligned}$$

with

6 $$\begin{aligned} \eta _i= & {} -\frac{1}{3} \left( x_1- 2\sqrt{x_1^2-3 x_2} \cos \left[ \mu +\frac{2\pi }{3}(i-1)\right] \right) ,\nonumber \\ \mu= & {} \frac{1}{3} \arccos \bigg [\frac{9 x_1 x_2-2x_1^2-27x_3}{2(x_1^2-3x_2)^{3/2}}\bigg ], \ \ \text {with} \qquad x_1=-i \delta +\frac{1}{2}\big (\gamma +\kappa _c+\kappa _m\big ),\nonumber \\ x_2= & {} 2 \lambda _1^2+\lambda _2^2+\frac{1}{4} \big (\gamma \kappa _c+\kappa _c \kappa _m+\gamma \kappa _m\big )-\frac{i}{2} \delta \big (\gamma + \kappa _m\big ), \nonumber \\ x_3= & {} \lambda _1^2 \kappa _m+\frac{1}{2} \gamma \lambda _2^2+\frac{1}{8} \gamma \kappa _c \kappa _m-\frac{i}{4}\gamma \delta \kappa _m. \end{aligned}$$

Now, we use the density operator for both the qubit system and the field–magnon system. After evaluating the sub-trace for the density operator of the system using Eq. (4), we get the reduced density operator for the two-qubit subsytem as

7 $$\begin{aligned} \hat{\rho }_{qubit}= & {} Tr_{c,m}(|\psi \rangle \langle \psi |)\nonumber \\= & {} |c_3(t)|^2|eg \rangle \langle eg|+ |c_4(t)|^2|ge \rangle \langle ge|\nonumber \\{} & {} + (|c_1(t)|^2+|c_2(t)|^2)|gg \rangle \langle gg|+ c_3(t)c_4^*(t) |eg \rangle \langle ge|+c_3^*(t)c_4(t) |ge \rangle \langle eg|. \end{aligned}$$

Likewise, the reduced density operator for the field–magnon subsystem is given by

8 $$\begin{aligned} \hat{\rho }_{c,m}= & {} Tr_{qubit}(|\psi \rangle \langle \psi |)\nonumber \\= & {} (|c_3(t)|^2+|c_4(t)|^2)|00 \rangle \langle 00|+|c_1(t)|^2|01 \rangle \langle 01|+ |c_2(t)|^2 |10 \rangle \langle 10|\nonumber \\{} & {} +c_1(t)c_2^*(t) |01 \rangle \langle 10|+c_1^*(t)c_2(t) |10 \rangle \langle 01|. \end{aligned}$$

Hereinafter, we need to reconstruct the steering inequality of two distinct mode fields and two-qubit via probabilities for joint measurements and LHS state.

## Steering inequality

We employ the LHS models needed that probabilistic for joint measurement at various labs *A* (Alice) and *B* (Bob), which is presented in^51^, to get the steering inequalities for any arbitrary bipartite observables

9 $$\begin{aligned} P(X_A,X_B|x_A,x_B,\kappa )=\int d\lambda P(\lambda |\kappa )P(X_A|x_A,\lambda ,\kappa )P(X_B|x_B,\lambda ,\kappa ), \end{aligned}$$

where $x_i$ identify the selected range of potential experiments, $X_j$ are the corresponding outcomes, $\kappa$ denotes the explicit characterization of the preparation procedure that is related to the experimenters, while $\lambda$ is used to label any possibally unknown variables that may have relevance to the experiments under consideration and for which a sufficient specification is needed.

Further, we assume that Alice’s local state is quantum. Specifically, there must exist a quantum state $\rho _{\lambda ,\kappa }$ such that it holds true for all outcomes $X_A$ of all measurements $x_A$

10 $$\begin{aligned} P(X_A|x_A,\lambda ,\kappa )=Tr[E_{X_A}\rho _{\lambda ,\kappa }]\equiv P_Q(X_A|x_A,\lambda ,\kappa ), \end{aligned}$$

where $E_{X_A}$ is the positive operator valued measure element associated with $X_A$. With this presumption and using the work of Wiseman et al.^51^, the EPR steering (of Alice’s state by Bob) occurs (leaving $\kappa$,$x_A$,$x_B$ henceforth implicit), and then

11 $$\begin{aligned} P(X_A,X_B)=\int d\lambda P(\lambda )P_Q(X_A|\lambda )P(X_B|\lambda ). \end{aligned}$$

Now, we employ the previous equation to get the cavity–magnon steering and qubit–qubit steering in the next two subsections.

### Cavity–magnon steering

Using Eqs. (9–11) and according to Refs.^52,53^, we define complex functions $F_j^{\pm }=X_j \pm i Y_j$ in terms of measurement outcomes $X_j,Y_j$ at each site $j\in \{1,2\}$. For any LHS (j,2) model (11), $\langle \prod _{i=1}^{2}F_j^{s_j}\rangle =\int d\lambda P(\lambda )\prod _{i=1}^{2}\langle F_j^{s_j}\rangle _\lambda$, where $s_j \in \{+,-\}$. Here $\langle F_j^\pm \rangle _\lambda =\langle X_j\rangle _\lambda \pm i \langle Y_j\rangle _\lambda$ with $\langle \bullet \rangle _\lambda =\sum _{\bullet _j}P(\bullet |\lambda )\bullet$, and $P(\bullet |\lambda )=P_Q(\bullet |\lambda )$ is obtained for trusted party mode. Thus the variance inequality reads^54^

12 $$\begin{aligned} \left| \left\langle \prod _{j=1}^{2}F_j^{s_j}\right\rangle \right| ^2\le \int d\lambda P(\lambda )\prod _{j=1}^{2}|\langle F_j^{s_j}\rangle _\lambda |^2. \end{aligned}$$

Here, $|\langle F_j^{\pm }\rangle _\lambda |^2=\langle X_j\rangle _\lambda ^2+\langle Y_j\rangle _\lambda ^2$, Considering that variances are non-negative, the following is true for any LHS (untrusted) state: $|\langle F_j^{\pm }\rangle _\lambda |^2\le \langle X_j^2\rangle _\lambda +\langle Y_j^2\rangle _\lambda$. By utilizing the uncertainty principle to impose constraints on the trusted (local quantum) state, we can establish that $(\Delta X_1)^2+(\Delta Y_1)^2\ge C_1$, where $C_1$ is dependent on the operators associated with $x_1$ and $y_1$. Through substitution into Eq. (12), we can achieve a range of non-locality criteria, which can be expressed as the following inequality^51^

13 $$\begin{aligned} \left| \left\langle \prod _{j=1}^{2}F_j^{s_j}\right\rangle \right| ^2\le \langle (X_1^2+Y_1^2-C_1)(X_2^2+Y_2^2) \rangle . \end{aligned}$$

The measurements $x_1$ and $y_1$ have not yet been subjected to any assumptions. For the continuous case, we assume position–momentum conjugation relations $[x_1,y_1]=i$ for the trusted site, in which the local uncertainty relation $(\Delta X_j)^2+(\Delta Y_j)^2\ge 1$,i.e., $C_1=1$ is implied. Thus the LHS model (13) implies

14 $$\begin{aligned} \left| \left\langle \prod _{j=1}^{2}F_j^{s_j}\right\rangle \right| ^2\le \langle (X_1^2+Y_1^2-1)(X_2^2+Y_2^2) \rangle . \end{aligned}$$

With two trusted parties, we obtain the entanglement criterion of Hillery and Zubairy^55^; with two untrusted parties, we obtain the Bell inequality of Cavalcanti et al.^53^. We now show how these inequalities can be violated using quantum mechanics. Using quadrature operators $\hat{x}_j=(\hat{a}_j+\hat{a}_j^\dagger )/\sqrt{2}$, $\hat{y}_j=i(\hat{a}_j-\hat{a}_j^\dagger )/\sqrt{2}$, where $\hat{a}_j^\dagger$, $\hat{a}_j$ are bosonic operators satisfying $([\hat{a}_j,\hat{a}_k^\dagger ]=\delta _{i,k})$, we obtain $F_j^+=\sqrt{2}\hat{a}_j^\dagger$ and $F_j^-=\sqrt{2}\hat{a}_j$, and $(\hat{x}_j^2+\hat{y}_j^2)=2\hat{a}_j^\dagger \hat{a}_j+1=2\hat{n}_j+1$, $\hat{n}_j$ being the number operator for each site. Now the inequality (14) may be violated at

15 $$\begin{aligned} |\langle \hat{a}_i\hat{a}_j^\dagger \rangle |^2>\langle \hat{n}_i(\hat{n}_j+1/2) \rangle , (i\ne j), i,j \in \{1,2\}. \end{aligned}$$

Consequently, the cavity–magnon steering measurement when the field ‘j’ steers the field ‘i’ is defined by

16 $$\begin{aligned} S_{ij}=|\langle \hat{a}_i\hat{a}_j^\dagger \rangle |^2-\langle \hat{a}_i^\dagger \hat{a}_i (\hat{a}_j^\dagger \hat{a}_j+\frac{1}{2})\rangle >0. \end{aligned}$$

Now, we apply (16) in (8) when the magnon mode steers the cavity–field, is

17 $$\begin{aligned} S_{cm}= & {} \max \big \{0, |\langle \hat{m}\hat{c}^\dagger \rangle |^2-\langle \hat{c}^\dagger \hat{c}(\hat{m}^\dagger \hat{m}+\frac{1}{2})\rangle \big \}\nonumber \\= & {} \max \big \{0,|c_2(t)|^2|c_1(t)|^2-\frac{1}{2}|c_2(t)|^2\big \}. \end{aligned}$$

Likewise, when the cavity–field steers the magnon mode according to

18 $$\begin{aligned} S_{mc}= & {} \max \big \{0,|\langle \hat{m}\hat{c}^\dagger \rangle |^2-\langle \hat{m}^\dagger \hat{m} (\hat{c}^\dagger \hat{c}+\frac{1}{2})\rangle \big \}\nonumber \\= & {} \max \big \{0,|c_2(t)|^2|c_1(t)|^2-\frac{1}{2}|c_1(t)|^2\big \}. \end{aligned}$$

**Figure 1:**
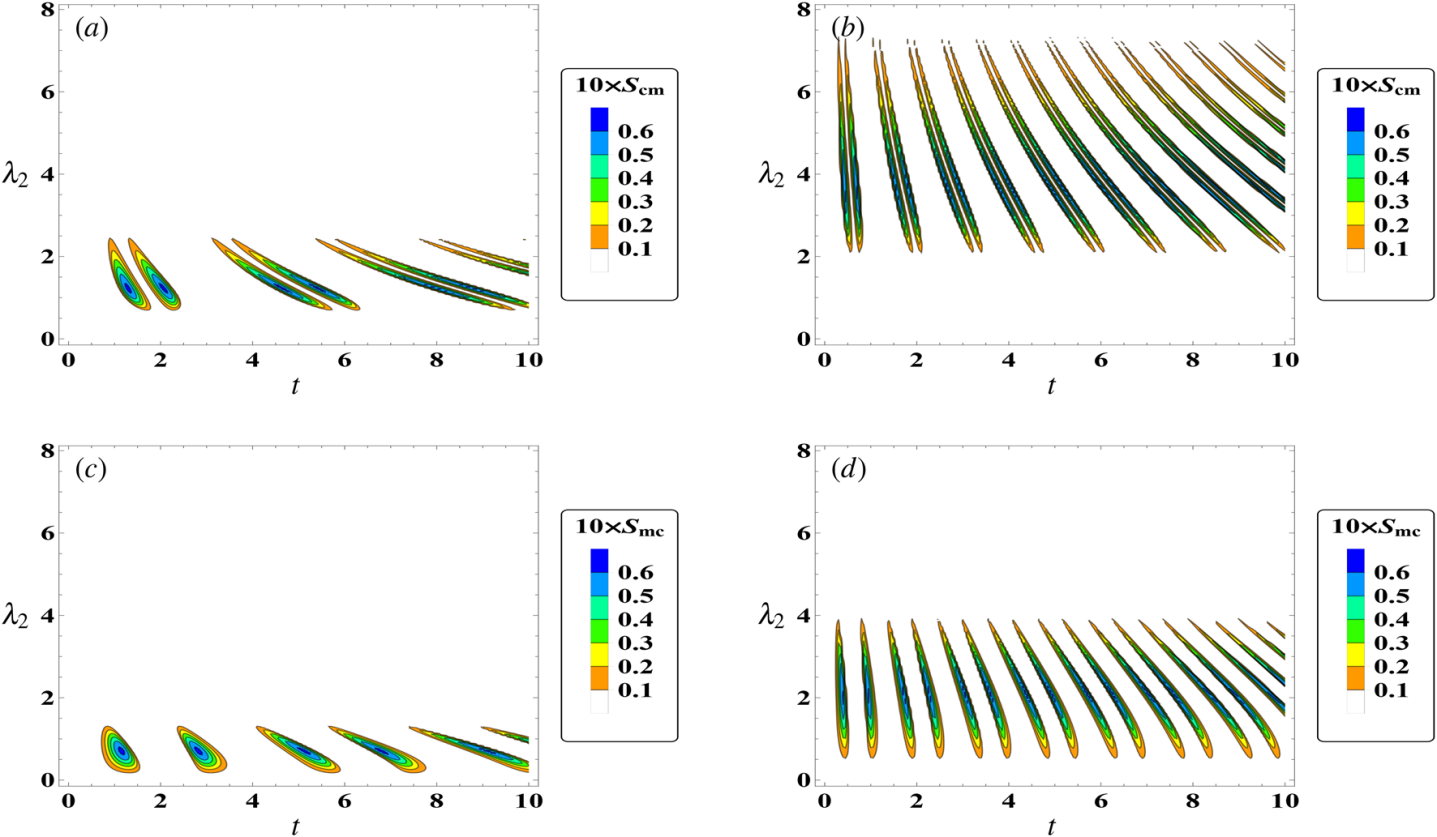
The steering behavior of $S_{cm}$ Eq. (17) in resonance case $\delta =0$ with $\gamma =0$, $\kappa _c=0$, and $\kappa _m=0$. (**a**) $\lambda _1$ = 1, (**b**) $\lambda _1$ = 3. (**c**,**d**) display the $S_{mc}$ Eq. (18) with same parameter as (**a**,**b**), respectively.

Figure 1 displays the contour behaviour of magnon–field and field–magnon steering in ($t, \lambda _2$) plane. We assume that the system in the resonance case ($\delta =0$) and the damping parameters are equal to zero, while we take various values of effective coupling of atom–field ($\lambda _1$). The results depicted in Fig. 1a illustrate that, for a weak coupling of atom–field ($\lambda _{1}=1$), the magnon-to-cavity steering achieved at small values of $\lambda _2$. However, increasing the value of $\lambda _1$ (as shown in Fig. 1b) significantly shifts the steering function $S_{cm}$ towards higher values of $\lambda _2$ , and number of peaks increased. Besides, the steering $S_{cm}$ is presented in Fig. 1c for a small value of the coupling parameter $\lambda _1=1$, where it is observed that the steering area in (*t*, $\lambda _2$) located at $0<\lambda _2<2$. As the atom–field coupling parameter ($\lambda _1=3$) increases (Fig. 1d), the steering function $S_{mc}$ expands along the $\lambda _2$-axis, resulting in $0<\lambda _2<4$, and the number of oscillations increases during the time of evolution.

Overall, Fig. 1 illustrates that both functions have identical upper bounds, with $S_{cm}$ and $S_{mc}$ both equal to 0.06. Moreover, the correlation between the magnon and the field is enhanced by the atom–field coupling parameter ($\lambda _1$), particularly when the magnon steers the field more effectively than vice versa. Notably, the steering area in the $(t, \lambda _2)$-plane for $S_{cm}$ is larger than that for $S_{mc}$. This observation may be attributed to the distribution of coherence between the measurement process for $S_{mc}$ and the interaction between the atoms and the field. The impact of magnon–cavity coupling on the steering between subsystems cannot be generalized across all quantum systems. It is important to consider the specific conditions of each quantum system, including its initial state and the duration of interaction, as well as the environmental factors that may impact it. However, when the two qubits are initially in a maximally entangled state, it can be said that a reduced effect of environmental coupling (the coupling between the qubits and the cavity) will maintain at least some level of partial entanglement between the atoms. In this case, increasing the coupling between the magnon and cavity results in strengthened entanglement between the two atoms. This suggests that the magnon has a strong attraction towards the cavity field. These references insure our results for some others different optomechanical system^56–58^.

**Figure 2:**
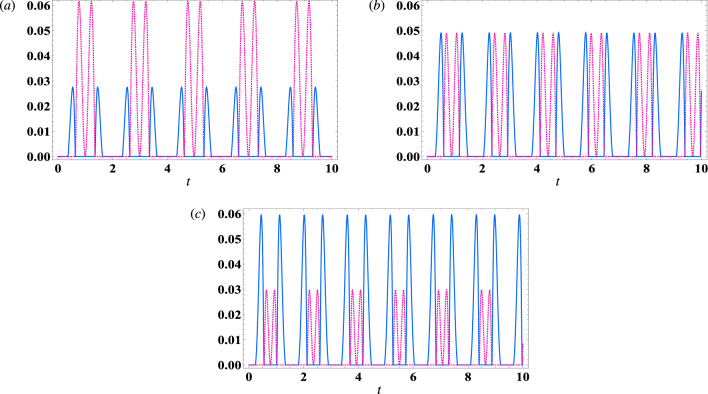
The steering behavior of $S_{cm}$ (dot-curve) and $S_{mc}$ (solid-curve) against the time *t* in the resonance case $\delta =0$ with $\gamma =0$, $\kappa _c=0$, $\kappa _m=0$, and $\lambda _{2}=2$. (**a**) $\lambda _1$ = $\sqrt{3}$, (**b**) $\lambda _1$ = $\frac{8}{1+\sqrt{8}}$, (**c**) $\lambda _1$ = $\sqrt{6}$.

Figure 2 displays the general behavior of the correlations $S_{cm}$ and $S_{mc}$ in the shared area illustrated in Fig. (1). The dotted curve represents the magnon mode’s ability to steer the cavity mode field, as indicated by $S_{cm}$. In contrast, the solid curve represents the cavity mode field’s ability to steer the magnon mode, as shown by $S_{mc}$. Notably, the steering behavior from the magnon mode to the cavity mode is not symmetrical to the steering behavior from the cavity mode to the magnon mode, with the positive values of the two functions being non-identical. In Fig. 2a, we decrease the effective coupling to $\lambda _1=\sqrt{3}$. It is evident that the upper bounds of $S_{cm}$ increase while $S_{mc}$ decreases. In Fig. 2b, we demonstrate the effect of effective couplings, with $\lambda _1=\frac{8}{1+\sqrt{8}}$ resulting in the two functions having equal upper bounds. Both functions exhibit periodic oscillations with time and sudden death in certain intervals. However, by increasing $\lambda _1$ to $\sqrt{6}$ (Fig. 2c), the upper bounds of $S_{cm}$ decrease while $S_{mc}$ increases. Based on the analysis, it can be inferred that enhancing the effective coupling $\lambda _1$ can result in a displacement of the upper limits of $S_{mc}$ ($S_{cm}$) towards (away from) the shared region of the two functions.

**Figure 3:**
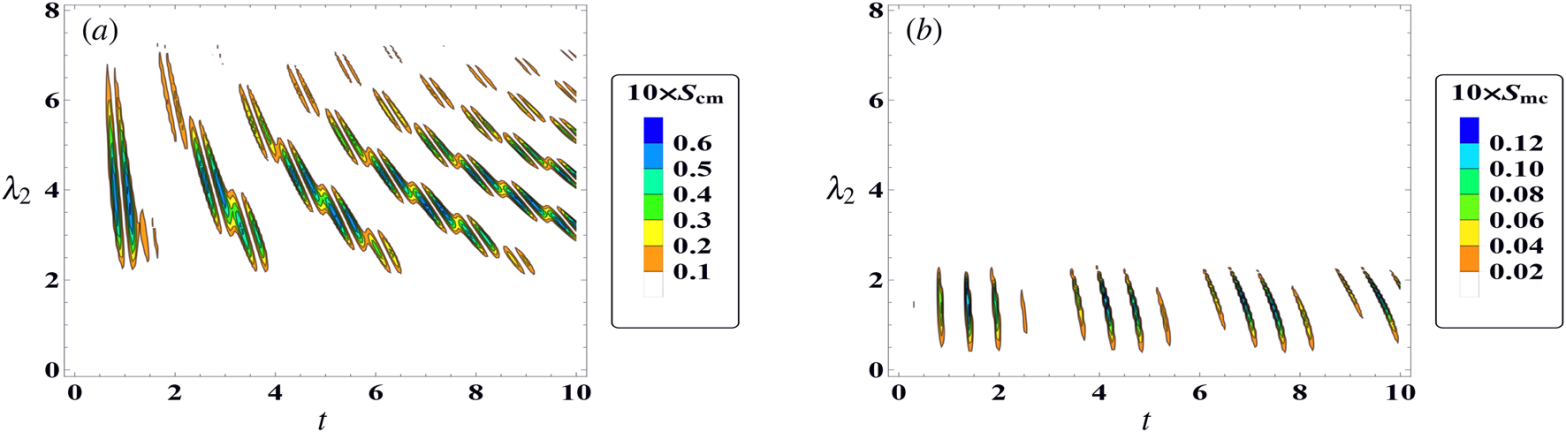
The steering behavior in the off-resonance case $\delta =7$, $\gamma =0$, $\kappa _c=0$, $\kappa _m=0$, and $\lambda _1$ = 3, where (**a**) $S_{cm}$, (**b**) $S_{mc}$.

The effect of off-resonance case $\delta =7$ on the behaviour of cavity–magnon steering with $\gamma =0$, $\kappa _c=0$, and $\kappa _m=0$, and $\lambda _1$ = 3 is displayed in Fig. 3. The results indicate that the maximum limit for the steering from magnon to cavity remains unchanged in the presence of detuning, as illustrated in Fig. 3a ($\max [S_{cm}]=0.06$). However, the regions of steering in the $(t, \lambda _2)$-plane undergo rearrangements. As the values of $\lambda _2$ and time increase, the steering peaks become separated and the number of peaks increasing accordingly. From the observations made in Fig. 3b, it can be inferred that the upper limits of the function $S_{mc}$ are reduced in the off-resonance scenario. This indicates that an increase in detuning can have an impact on the local measurement of the cavity, leading to an increase in decoherence and then a decrease in steering.

**Figure 4:**
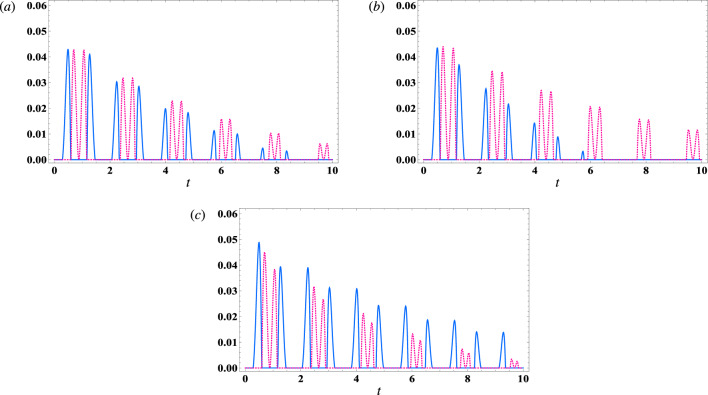
The steering behavior of $S_{cm}$ (dot-curve) and $S_{mc}$ (solid-curve) against the time *t* in the resonance case $\delta =0$ with $\lambda _1$ = $\frac{8}{1+\sqrt{8}}$ , and $\lambda _{2}=2$. (**a**) $\gamma =0.1$, $\kappa _c=0$, $\kappa _m=0$, (**b**) $\gamma =0$, $\kappa _c=0.1$, $\kappa _m=0$ , (**c**) $\gamma =0$, $\kappa _c=0$, $\kappa _m=0.1$.

Now, we will analyse the impact of decay within the atomic subsystem, field subsystem, and magnon subsystem on the steering degree between the magnon mode and cavity mode field. Specifically, we will focus on the case where $\lambda _1=\frac{8}{1+\sqrt{8}}$ and $\lambda _2=2$ in the resonance condition. As depicted in Fig. 4a, the addition of atomic damping $\gamma =0.1$ ( $\kappa _c=0$, $\kappa _m=0$) to the system results in a decay of the steering functions as the interaction time increases. Notably, the decay rate of the $S_{mc}$ function is found to be faster compared to the $S_{cm}$ function. Additionally, Fig. 4b illustrates the effect of damping within the cavity mode subsystem $\kappa _c=0.1$ ($\gamma =0$, $\kappa _m=0$) on the steering degree between the magnon and cavity. It is observed that, as the interaction time increases, the steering degree decreases. Moreover, the decay of steering from the cavity to the magnon mode is faster than the decay of steering from the magnon mode to the cavity. In contrast, Fig. 4c demonstrates that the inclusion of magnon damping ($\kappa _m=0.1$, $\gamma =0$, $\kappa _c=0$) leads to a decay in the steering degree. However, it is important to note that $S_{cm}$ is less than $S_{mc}$ in this case.

### Qubit–qubit steering

From Eq. (11), it is important to consider that the probability density of measuring $X_A$ is derived from a single quantum state, specifically that of quantum system A, rather than B, whose preparatory details are entirely within the scope of control of the hidden variable $\lambda$. By following the findings of Walborn et al.^46^, a Local Hidden State Model (LHSM) can provide an explanation for continuous observables, where the continuous relative entropy between $P(X_A,\lambda |X_B)$ and $P(\lambda |X_B)P(X_A|X_B)$ for any pair of probability distributions or densities is greater than zero

19 $$\begin{aligned} h(X_A|X_B)\ge \int d\lambda p(\lambda ) h_Q(X_A|\lambda ). \end{aligned}$$

Here, $h(X_A|X_B)$ refers to the continuous Shannon entropy that arises from the probability density resulting from $P_Q(X_A|\lambda )$. Upon creating steering inequalities for random observables, we observe that the same arguments used to establish Local Hidden State (LHS) constraints for continuous observables can also be applied to discrete observables. For instance, let $R^A$ and $S^A$ be discrete observables with corresponding N eigenstates ${R^A_i}$ and ${S^A_i}$, $i\in {1,\dots ,N}$, and let ${R^B}$ and $S^B$ be the corresponding observables for system B. As the relative entropy is positive for both continuous and discrete variables, we can establish a matching LHS constraint for discrete observables, which reads^59^

20 $$\begin{aligned} H(R_A|R_B)\ge \sum _\lambda P(\lambda )H_Q(R_A|\lambda ), \end{aligned}$$

where $H_Q(R_A|\lambda )$ is the discrete Shannon entropy of the probability distribution $P_Q(R_A,\lambda )$. By taking the weighted average of these entropies using the weight function $P(\lambda )$, we can easily obtain the right-hand side of Eq. (19). For every pair of continuous observables with an entropic uncertainty relation, the corresponding steering satisfies the folowing inequality

21 $$\begin{aligned} h(x^A|x^B)+h(k^A|k^B)\ge log(\pi e), \end{aligned}$$

where $\hat{x}$ are continuous observables and $\hat{k}$ is wave number. Based on the entropic uncertainty relation, it is possible to express an uncertainty relation for any pair of discrete observables $\hat{R}$ and $\hat{S}$ that share the same N-dimensional Hilbert space and discrete eigenstates $|R_i\rangle$ and $|S_i\rangle$, respectively. This relation is formulated as follows^60^

22 $$\begin{aligned} H_Q(R)+H_Q(S)\ge log(\Omega ), \ \text {with} \quad \Omega \equiv \min _{i,j}\bigg \{\frac{1}{|\langle R_i|S_i\rangle |^2}\bigg \}. \end{aligned}$$

For pairs of discrete observables using the discrete entropic uncertainty relation (22) and LHS restraint for discrete observables (20), we can rewrite the entropic steering inequality as

23 $$\begin{aligned} H(R^A|R^B)+H(S^A|S^B)\ge log(\Omega ^A), \end{aligned}$$

where $\Omega ^A$ is the value $\Omega$ assigned to the observables $R^A$ and $S^A$.

Recent experimental studies^61,62^ have demonstrated the validity of the EPR-steering inequality for entangled photons’ discrete and continuous components of position and momentum variables. It is essential to note that an EPR-steering inequality exists for every entropic uncertainty relation, including those that connect more than two observables. Sanchez-Ruiz^63^ proposed entropic uncertainty relations for complete sets of pairwise complementary (mutually unbiased) observables $R_i$, where $i\in {1,\ldots ,N }$, and when N is even, the uncertainty relation is formulated as follows

24 $$\begin{aligned} \sum _{k}^{N+1}H(R^A_k|R^B_k)\ge \left( \frac{N}{2}\log _2{\left( \frac{N}{2}\right) }\right) +\left( \left( 1+\frac{N}{2}\right) \log _2{\left( 1+\frac{N}{2}\right) }\right) , \end{aligned}$$

where $H(B|A) = H(\hat{\rho }_{AB}) - H(\hat{\rho }_A)$ is the conditional Shannon entropy of an arbitrary bipartite quantum system $\rho _{AB}$ in *N* even dimensional systems. In two dimensional subsystems ($N=2$), one may employ the Pauli matrices as measurements. Hence, the qubit–qubit EUR steering inequality reads

25 $$\begin{aligned} H(\sigma _x^B|\sigma _x^A)+H(\sigma _y^B|\sigma _y^A)+H(\sigma _z^B|\sigma _z^A)\ge 2, \end{aligned}$$

where $H(\sigma _i^B|\sigma _i^A)=H(\rho _{AB})_i- H(\rho _A)_i$, and *i* related to Pauli spin operators. In which, $H(\rho _{AB})_i=- \sum _{n,m=1}^{2} P_i^{n,m} \log _2 P_i^{n,m}$, $P_i^{n,m}=\langle \phi ^i_n, \phi ^i_m|\rho _{AB}|\phi ^i_n, \phi ^i_m \rangle$, where the states $|\phi ^i_j \rangle$ represent the two possible eigenvectors of $\sigma _i$. Likewise, for the reduced density state $\rho _{A}=Tr_B[\rho _{AB}]$, the information entropy gives $H(\rho _A)_i=- \sum _{n}^{2} P_i^{n} \log _2 P_i^{n}$, with $P_i^{n}= \langle \phi ^i_n |\rho _{A}|\phi ^i_n \rangle$, (for more details, see^64,65^). Using previous notation and employing the qubit–qubit density operator (7), the qubit–qubit EUR-steering inequality can be given by

26 $$\begin{aligned} I_{AB} =&2[(1+Re(u))\log _2{(1+Re(u))}+(1-Re(u))\log _2{(1-Re(u))}]\nonumber \\&-(1+v)\log _2{(1+v)}- (1-v)\log _2{(1-v)}+\nonumber \\&+\frac{1}{2}[(1+w+v+s)\log _2{(1+w+v+s)}\nonumber \\&+(1+w-v-s)\log _2{(1+w-v-s)}+(1-w-v+s)\log _2{(1-w-v+s)}\nonumber \\&+(1-w+v-s)\log _2{(1-w+v-s)}]\le 2, \end{aligned}$$

with

27 $$\begin{aligned} u&=2c_3(t)c_4^*(t), \end{aligned}$$

28 $$\begin{aligned} v&=-|c_3(t)|^2+|c_4(t)|^2-|c_1(t)|^2-|c_2(t)|^2, \end{aligned}$$

29 $$\begin{aligned} w&=-|c_3(t)|^2-|c_4(t)|^2+|c_1(t)|^2+|c_2(t)|^2, \end{aligned}$$

30 $$\begin{aligned} s&=|c_3(t)|^2-|c_4(t)|^2-|c_1(t)|^2-|c_2(t)|^2. \end{aligned}$$

Since $c_3(t)=c_4(t)$ implies that $v=s$, so the Atomic-Steering is symmetric ($I_{AB}=I_{BA}$).

The normalized qubit–qubit steering equations (26) can be written as^64^

31 $$\begin{aligned} \mathscr {S}_{A B}= \max \bigg \{0,\frac{I_{AB}-2}{I_{max}-2}\bigg \}, \end{aligned}$$

where $I_{max}=6$ is calculated for a maximally entangled two-qubit state (i.e. Bell states).

**Figure 5:**
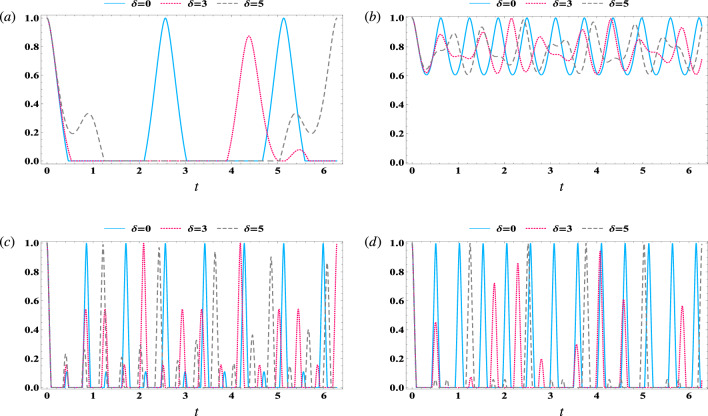
The behavior of qubit–qubit steering $\mathscr {S}_{A B}$ Eq. (31) for different values of detuning against the time *t*, where $\gamma =0$, $\kappa _c=0$, and $\kappa _m=0$. (**a**) $\lambda _1$ = 1, $\lambda _2$ = 2, (**b**) $\lambda _1$ = 1, $\lambda _2$ = 10, (**c**) $\lambda _1$ = 5, $\lambda _2$ =2, (**d**) $\lambda _1$ = 5, $\lambda _2$ = 10.

Figure 5 illustrates the impact of different effective coupling parameters, $\lambda _1$ and $\lambda _2$, on the qubit–qubit steering (Eq. 31) in both the resonance and off-resonance scenarios. The solid curve represents the resonance case, while the dotted (dashed) curve represents the lesser (larger) value of the off-resonance case. For weak couplings ($\lambda _1 =1$ and $\lambda _2=2$) and the resonance case (solid curve), Fig. 5a indicates that the function $\mathscr {S}_{AB}$ exhibits periodic oscillations between its upper and lower limits. In the off-resonance cases ($\delta$ = 3), the function $\mathscr {S}_{AB}$ displays chaotic oscillations, leading to a decrease in steering between the two qubits. More increasing in the detuning parameter ($\delta$ = 5) results in longer unsteerable periods as time progresses. However, at the onset of interaction, steering is at its maximum (Bell state). In Fig. 2b, the impact of the magnon–cavity coupling increase on the steering $\mathscr {S}_{AB}$ is examined. The findings indicate a significant improvement in the steering, with no unsteerable periods and a lower limit of $\mathscr {S}_{AB}$ greater than 0.6. Furthermore, the semi-cyclical oscillation of the steering is re-adjusted by $\lambda _2$, even with an increase in $\delta$. It is evident that augmenting $\lambda _1$ results in an increase in the number of oscillations, which subsequently elevates the number of occurrences of reaching the maximum value. Additionally, this leads to an increase in the randomness of steering with a corresponding increase in detuning (refer to Fig. 5c). On the other hand, significant values of coupling $\lambda _2$ reorganize the randomness generated by the escalation of $\lambda _1$, even with large values of detuning (as depicted in dashed curve in Fig. 5d).

**Figure 6:**
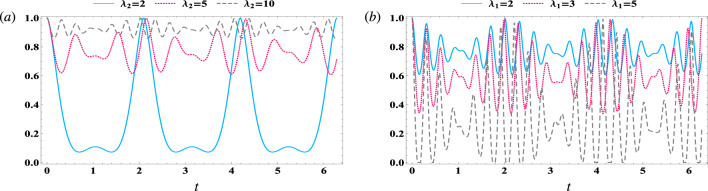
The behavior of qubit–qubit steering $\mathscr {S}_{A B}$ Eq. (31) against the time *t* in the off-resonance case $\delta$ = 3, where $\gamma =0$, $\kappa _c=0$, and $\kappa _m=0$. (**a**) $\lambda _1$ = 1 , (**b**) $\lambda _2$ = 20.

In the absence of the damping parameters, Fig. 6 examines the impact of various effective couplings ($\lambda _1$, and $\lambda _2$) on qubit–qubit steering $\mathscr {S}_{AB}$ for the off-resonance scenario ($\delta =3$). Figure 6a demonstrates the effect of different field–magnon coupling values at a fixed coupling of $\lambda _1=1$. The results indicate that an increase in $\lambda _2$ results in an improvement in steering, where the lower limits of $\mathscr {S}_{AB}$ increase, and the number of oscillations rises. This can be attributed to the fact that increasing $\lambda _2$ may enhance the coherence between the cavity and the magnon, thereby providing more opportunities for robust bonding between the two qubits. Conversely, Fig. 6b illustrates the increase in atom–field coupling with varying values and a greater magnitude of magnon–field coupling. The findings indicate that as $\lambda _1$ increases, the qubit–qubit steering decreases. This can be attributed to the fact that the interaction with the cavity field may elevate the decoherence of the two-qubit system.

**Figure 7:**
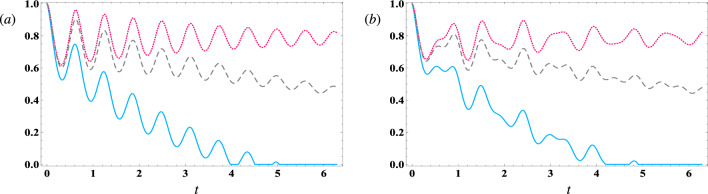
The behavior of qubit–qubit steering $\mathscr {S}_{A B}$ Eq. (31) against the time *t* with $\lambda _1$ = 1, and $\lambda _2$ = 10, where $\gamma =0.1$, $\kappa _c=0$, $\kappa _m=0$ (solid curve), $\gamma =0$, $\kappa _c=1$, $\kappa _m=0$ (dotted curve), and $\gamma =0$, $\kappa _c=0$, and $\kappa _m=1$ (dashed curve). (**a**) $\delta$ = 0, (**b**) $\delta$ = 3.

The effects of three types of damping (atomic, cavity field, and magnon) on quantum steering between two qubits are depicted in Fig. 7. We have let in this case $\lambda _1=1$, $\lambda _2=10$, and we analyzed under both in the resonance case (Fig. 7a) and off-resonance case (Fig. 7b). Introducing atomic damping (solid curves) significantly deteriorates the inter-qubit steering, causing it to decay over time. Notably, the decay rate observed in the resonance case is greater than that observed in the off-resonance case. Furthermore, when large values are assigned to the damping parameters, whether in the cavity field (dotted curves) or the magnon mode (dashed curves), the steering between the two qubits is diminished. However, it is important to note that the value of atomic steering in the presence of the field damping rate exceeds that observed when the effect of magnon damping is added. This discrepancy can be explained by the strong coupling between the cavity and the magnon. Additionally, as time progresses, the atomic steering in both the resonance and off-resonance cases diminishes to residual values.

## Conclusion

This study delves into the feasibility of steering between two parties who share a temporal state generated from a system comprising a two-qubit state and magnon mode within a cavity mode field. It is postulated that the two-qubit state is initially prepared in a maximally entangled state, while the magnon mode and the cavity are in the vacuum state. The inequalities of cavity–magnon and qubit–qubit EPR steering are explored. Furthermore, the impact of the detuning parameter and the effective coupling of the atom–field, magnon–field, and damping parameters is investigated.

In the event where the magnon mode steers the cavity–field, the overall behaviour is asymmetric when the cavity steers the magnon. Despite the initial separability of the field and the magnon, a weak quantum steering is generated between them proportional to the strength of their coupling. Notably, the possibility of steering from the magnon to the cavity is greater than steering from the cavity to the magnon. An increase in the atom–field coupling augments the bidirectional steering between the cavity and magnon. Conversely, the detuning parameter diminishes the field steering. Atomic decay prominently affects quantum steering between the optical cavity field and magnon mode. Cavity field decay leads to appreciable deterioration of the steering from the field to the magnon mode over time. Conversely, applying magnon decay causes rapid attenuation of the magnon-to-field steering, significantly reducing this field steering correlation. The directional asymmetry in how the different decay mechanisms influence quantum steering can be attributed to the fact that field/magnon decay parameter acts directly on the cavity field/magnon mode subsystem itself.

The impact of the three parameters on atomic steering is markedly distinct. The detuning parameter diminishes atomic steering, while augmenting it in the presence of magnon–field coupling enhanced and reorganized the stochastic behaviour arising from increasing atomic-field coupling. Generally, coupling of the atom–field reduced steering and induced random behaviour due to a reduction in correlation between the two atoms. Conversely, magnon–field coupling yielded a significant improvement in atomic steering. The entry of the qubit decay parameter significantly deteriorates the quantum steering between the two qubits. In contrast, even substantial decay rates of cavity field and magnon mode have a relatively minor impact on the inter-qubit steering. This suggests the atomic decay mechanisms directly acting on the qubit subsystem itself dominate over the indirect effects of field and magnon dissipation.

In conclusion, the atomic system is superior to the field system for steering between two parties. It is recommended to increase magnon–field coupling, decrease atom–field coupling and decreasing the decay rates of subsystems.

## Data Availability

The used code of this study is available from the corresponding author upon reasonable request.
